# Integrative analysis of the multi-omics reveals the stripe rust fungus resistance mechanism of the *TaPAL* in wheat

**DOI:** 10.3389/fpls.2023.1174450

**Published:** 2023-06-05

**Authors:** Rong Liu, Xue Lv, Xiaohua Wang, Li Yang, Jun Cao, Ya Dai, Wang Wu, Yu Wu

**Affiliations:** ^1^ Faculty of Agriculture, Forestry and Food Engineering of Yibin University, Yibin, China; ^2^ Chengdu Institute of Biology, Chinese Academy of Sciences, Chengdu, China; ^3^ Wuhan Metware Biotechnology, Wuhan, Wuhan, China

**Keywords:** wheat, stripe rust, lignin, TaPAL, CYR34

## Abstract

Wheat is one of the major food crops in the world. However, stripe rust fungus significantly decreases wheat yield and quality. In the present study, transcriptomic and metabolite analyses were conducted in R88 (resistant line) and CY12 (susceptible cultivar) during Pst-CYR34 infection due to the limited availability of information regarding the underlying mechanisms governing wheat–pathogen interactions. The results revealed that Pst infection promoted the genes and metabolites involved in phenylpropanoid biosynthesis. The key enzyme gene *TaPAL* to regulate lignin and phenolic synthesis has a positive resistance contribution to Pst in wheat, which was verified by the virus-induced gene silencing (VIGS) technique. The distinctive resistance of R88 is regulated by the selective expression of genes involved in the fine-tuning of wheat–Pst interactions. Furthermore, metabolome analysis suggested that lignin biosynthesis-related metabolite accumulation was significantly affected by Pst. These results help to elucidate the regulatory networks of wheat–Pst interactions and pave the way for durable resistance breeding in wheat, which may ease environmental and food crises around the world.

## 1 Introduction

Plants are unceasingly under attack by pathogenic microbes (or pathogens) that have activated an additional plant immune system to defend against invading pathogens (Harvey et al., 2020). Stripe or yellow rust (Yr) of wheat, caused by *Puccinia striiformis* f. sp. *tritici* (Pst), is the most globally devastating fungal disease, which has massively decreased wheat production and quality and ranged from 10% to 70% in most of the wheat-growing areas (Kiran et al., 2017; Wu et al., 2022). In China, stripe rust is usually threatening major wheat-growing regions in the Northern, Southwestern, and Northwestern provinces along the Huai and Yellow River regions (Chen, 2005; Han et al., 2015; Jiang et al., 2022). Studies have found that the most effective and environment-friendly way to control wheat stripe rust is the cultivation of resistant varieties (Zheng et al., 2017; Chen et al., 2019), which can reduce the indiscriminate use of fungicides that cause serious environmental problems and health hazards to animals and humans. Wheat cultivars with race-specific resistance maintain resistance for only a few years due to the rapid evolution of new Pst races (Xu et al., 2019). For example, a new Pst race (V26, CYR34) virulent to the resistance gene *Yr26* has spread in several major wheat-growing regions where Yr26 is widely used in wheat breeding programs (Han et al., 2015). A recent study has reported that most current wheat cultivars and breeding lines (76%) are susceptible to Pst-CYR34 (Liu et al., 2010). Therefore, it is necessary to explore the mechanism of disease resistance and develop new strategies to improve disease resistance in wheat.

Certain disease-resistance-related enzymes and genes, including *PAL*, *peroxidase* (*POD*), and lignin, have been reported to play an important role in protecting plants against pathogen infection (Dias et al., 2017; Jamieson et al., 2017; Singh and Jha, 2017). It is well known that the phenylalanine ammonia-lyase (PAL) is usually involved in plants under biotic and abiotic stresses (Cass et al., 2015; Jamieson et al., 2017). Previous reports found that the *PAL* gene is considered to be involved in the main resistance processes in wheat (Van Eck et al., 2010). The enzymes of POD catalyze the polymerization of monolignols to generate lignin, which is an aromatic heteropolymer synthesized by phenylpropanoid metabolism (Zhang et al., 2020). The current perspective on plant immunity has evolved towards a comprehensive view of plant–pathogen interactions (Dodds and Rathjen, 2010). However, the gene expression profiles associated with the responses to stripe rust races in both resistant and susceptible wheat are still lacking, and most of the regulatory networks and the molecular mechanisms of the resistant gene to Pst remain unknown.

Therefore, in this study, the metabolomic and transcriptomic responses of wheat cv. Chuanyu12 (CY12) and the resistance breeding line R88 were investigated under Pst-CYR34 infection. In order to investigate the gene expression and metabolite accumulation in Pst response pathways and reveal the underling mechanisms governing wheat resistance to Pst, the candidate gene *TaPAL* was cloned and validated by virus-induced gene silencing (VIGS)-induced gene transient silencing, which proved that *TaPAL* plays an important role in wheat to Pst resistance.

## 2 Materials and methods

### 2.1 Plant materials and fungal treatment

The wheat cv. CY12 and the resistance breeding line R88 were used in this study. With the emergence of new Pst races, CY12 is highly susceptible to the currently predominant stripe rust. Wheat line R88 is an elite wheat germplasm from the offspring of CY12 with high all-stage resistance to CYR32 and CYR34. Artificial inoculation was conducted under controlled greenhouse conditions at the Gansu Academy of Agricultural Sciences at the wheat seedling stage. The wheat cv. Mingxian169 was used to monitor inoculation efficiency. The wheat leaves of CY12 and R88 were collected at 0 h (a), 24 h (b), and 72 h (c) post-inoculation, respectively (each time point with three biological replicates) (Wang et al., 2019; Francesconi and Balestra, 2020). All leaf samples were frozen in liquid nitrogen and stored in a −80°C freezer for subsequent RNA isolation, transcriptome sequencing, and metabolome detection.

### 2.2 RNA isolation and cDNA library construction for RNA sequencing

Total RNA of wheat leaves was extracted using the mirVana miRNA Isolation Kit (Ambion) following the manufacturer’s protocol. Then, mRNA was broken into short fragments at a suitable temperature in a thermomixer. Purified fragmented mRNA was used to synthesize first- and second-strand cDNA. The libraries were constructed using the TruSeq Stranded mRNA LT Sample Prep Kit (Illumina, San Diego, CA, USA) according to the manufacturer’s instructions. Then, 18 cDNA libraries were sequenced on the Illumina sequencing platform (HiSeqTM 2500 or Illumina HiSeq X Ten), and 125 bp/150 bp paired-end reads were generated. Raw reads containing poly-N and low-quality reads were removed to obtain clean reads. All clean reads were used for subsequent analyses and mapped to the reference genome using HISAT2.

### 2.3 Differential expression genes, GO, and KEGG analysis

The fragments per kilobase of transcript per million mapped reads (FPKM) value of each gene was calculated, and the read counts of each gene were obtained by htseq-count. Differentially expressed genes (DEGs) were analyzed and identified using the DEGseq R package and selected with |log2 FC|>1 and Q<0.005. GO enrichment and Kyoto Encyclopedia of Genes and Genomes (KEGG) pathway enrichment analyses of DEGs were performed using R based on the hypergeometric distribution during CYR34 infection. GO classification was performed by mapping the relation between SwissProt and GO terms. The major signaling pathways involved in DEGs were enriched in the KEGG database (Kanehisa et al., 2008).

### 2.4 Metabolite extraction and HPLC-MS/MS analysis

The freeze–dried wheat leaf samples were crushed and extracted with 1.0 ml of 70% methanol aqueous solution overnight at 4°C. The sample extracts were analyzed using a liquid chromatography electrospray ionization tandem mass spectrometry (LC-ESI-MS/MS) system (Q TRAP, Applied Biosystems API 6500 Q TRAP LC/MS/MS System, Foster City, CA, USA). The analytical conditions were quantified as previously described (Jost et al., 2019). Metabolites were quantified by calculating the area of each individual peak, and significant differences in content were set with thresholds of variable importance in projection ≥1 and fold change ≥1.5 or ≤0.5. Three biological and three technical replicates were performed in the metabolomics analysis.

### 2.5 Determination of lignin content and enzyme activity

R88 and CY12 wheat leaves were collected at 0, 24, and 72 hpi (Wang et al., 2019; Francesconi and Balestra, 2020). The lignin content was determined by the lignin-thioglycolic acid reaction (dos Santos et al., 2015). The determination of PAL enzyme activity was performed according to a previous study (Wang et al., 2016). The enzyme activities of POD and catalase (CAT) were measured by the increase in absorbance at 470 and 240 nm, respectively, according to the methods of Liu et al. (2019). Lipid peroxidation was evaluated by measuring malondialdehyde content (MDA). According to the method of the previous study (Liu et al., 2019), MDA content and proline (Pro) concentration were also determined with a slightly modified method.

### 2.6 qRT-PCR analysis

qRT-PCR was performed with a SYBR Green PCR kit (Qiagen, 204054) according to the manufacturer’s instructions. The experimental conditions were set as follows: 45 cycles at 95°C for 20 s, 55°C for 20 s, and 72°C for 20 s. All genes were subjected to three technical repetitions in this study. The mRNA expression level of the genes was calculated with the 2^−ΔΔCt^ method, and *GAPDH* was used as the internal reference gene. Each wheat leaf sample was repeated three times. The correlation between the RNA-seq and qRT-PCR results was analyzed using the R package version 3.1.3 (http://cran.r-project.org/). The normalized values of relative expression and FPKM values were calculated using log2 (fold change) measurements.

### 2.7 TaPAL gene cloning and BSMV-mediated gene silencing

Based on the gene sequence from the wheat genome (IWGSC v1.1), the *TaPAL* homologous genes (*TraesCS6A02G222900*, *TraesCS6B02G258400*, and *TraesCS6D02G212200*) of wheat were amplified from the cDNA of R88 and CY12 by three pairs of gene-specific primers (**Supplementary Table S1**), followed by cloning into the pEASY-T1 simple cloning vector (Transgene Biotech, Beijing, China) before sequencing. PCR was carried out as follows: initial denaturation at 98°C for 2 min, followed by 35 cycles of 98°C for 10 s, 52°C for 30 s, and 72°C for 1 min, followed by a final extension at 72°C for 5 min, followed by a 4°C hold. The purified PCR product was sequenced by Tsingke Co., Ltd. (www.tsingke.net).

Barley stripe mosaic virus (BSMV), consisting of RNAs α, β, and γ, induced gene silencing was conducted in this study. BSMV construct vectors were obtained from Professor Jie Liu’s lab at Northwest Agriculture & Forestry University. The 221 bp (V-TaPAL1as) and 202 bp (V-TaPAL2as) TaPAL gene fragments were amplified with PacI and NotI restriction sites using the primer pair V-TaPAL F/R (**Supplementary Table S1**) and inserted into the original BSMV:γ vector to produce the recombinant plasmid BSMV : TaPAL. Using the same method, we constructed the BSMV : TaPDS expression vector as the positive control. Plasmids BSMV : TaPAL, BSMV : TaPDS, and BSMV:γ were linearized followed by transcribing and capping *in vitro* using the RiboMA Large-Scale RNA Production System-T7 (Promega, USA) according to the manufacturer’s protocol. The BSMV:α, BSMV:β, and BSMV:γ, BSMV : TaPDS, and BSMV : TaPAL were separately mixed in a 1: 1: 1 ratio and then added to the FES buffer for mechanically rubbed inoculation in the dark for 24 h. Ten days after BSMV inoculation, the phenotype of the fourth leaves of wheat caused by Pst infection was measured. All primers used for gene cloning, vector construction, and qRT-PCR analysis are listed in **Supplementary Table S1**.

## 3 Results

### 3.1 Analysis of physiological indexes in wheat after Pst infection

**Figure 1:**
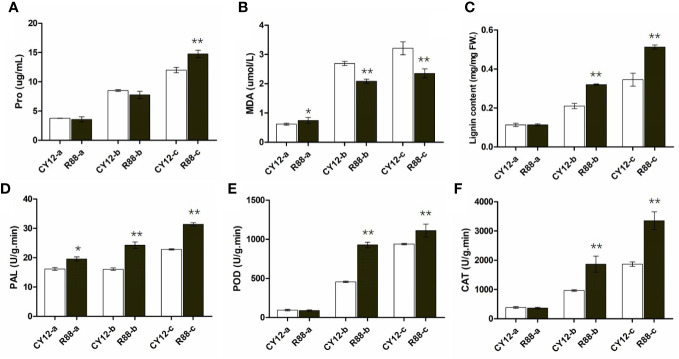
The physiological indices of the susceptible and resistant wheat seedlings were detected at different time points before and after Pst inoculation. Proline content **(A)**, malondialdehyde (MDA) content **(B)**, total lignin content **(C)**, and PAL, POD, and CAT enzyme activities **(D–F)** in wheat leaves. In each bar chart, a, b, and c represent 0 h before inoculation, 24 h after inoculation, and 72 h after inoculation, respectively. ** on the bar chart indicated the significant differences between R88 and CY12 after CYR34 inoculation at each time point (P<0.01).

Proline (Pro), malondialdehyde (MDA), lignin, PAL, POD, and CAT play important roles in plant response to biotic and abiotic stresses. Therefore, in this experiment, these physiological and biochemical indices were detected at different time points of wheat infection with CYR34 (**Figure 1**). The results showed that these physiological indexes were significantly increased in CY12 and R88 with the increase in Pst infection time. There was no significant change in proline content in both the 0- and 24-h Pst infections at CY12 and R88. Compared with CY12, the proline content of R88 increased significantly after 72 h of Pst treatment (**Figure 1A**). On the contrary, MDA content in R88 treated with Pst was significantly lower than that in CY12 (**Figure 1B**). In addition, the changes in lignin content, PAL, POD, and CAT enzyme activity were similar to those in CY12 and R88 at 24 and 72 h after inoculation with Pst. Among them, before inoculation (a), the basal lignin level, POD, and CAT enzyme activity levels of CY12 and R88 were similar; only PAL enzyme activity was significantly higher in R88 than in CY12 (**Figure 1**). At 24 h after Pst treatment (b), lignin, PAL, POD, and CAT increased significantly in R88 by 1.5, 1.5, 2.0, and 1.9 times, respectively. At 72 h after Pst treatment (c), lignin, PAL, POD, and CAT were significantly increased by 1.5, 1.4, 1.2, and 1.8 times in R88, respectively (**Figures 1C–F**). These results indicated that Pst-resistant wheat could significantly increase PAL, POD, and CAT enzyme activities, accumulate Pro and lignin content, and inhibit the increase in MDA to the maximum extent to protect its own cells from damage.

### 3.2 Transcriptome analysis and DEGs identification of wheat

To elucidate the molecular basis for the differential stripe rust response in CY12 and R88, comparative transcriptome analysis was conducted through RNA sequencing (RNA-seq). The 24- and 72-h Pst treatment time points were selected to investigate the wheat response to Pst-CYR34 at different infection stages. Eighteen cDNA libraries were characterized by Illumina HiSeq to detect the transcriptome level of gene expression information. After removing low-quality reads, clean reads were obtained, and more than 87% of the clean reads per library could be mapped to the wheat reference genome (IWGSC RefSeq V1.1). There are more than 60,000 transcripts with a length of 1,201–1,800 bp, and nearly 80,000 transcripts with a length of >1,800 (**Supplementary Figure S1A**). These transcripts were functionally annotated using the GO, KEGG, COG, NR, Swiss-Port, and Pfam databases (**Supplementary Figure S1B**).

**Figure 2:**
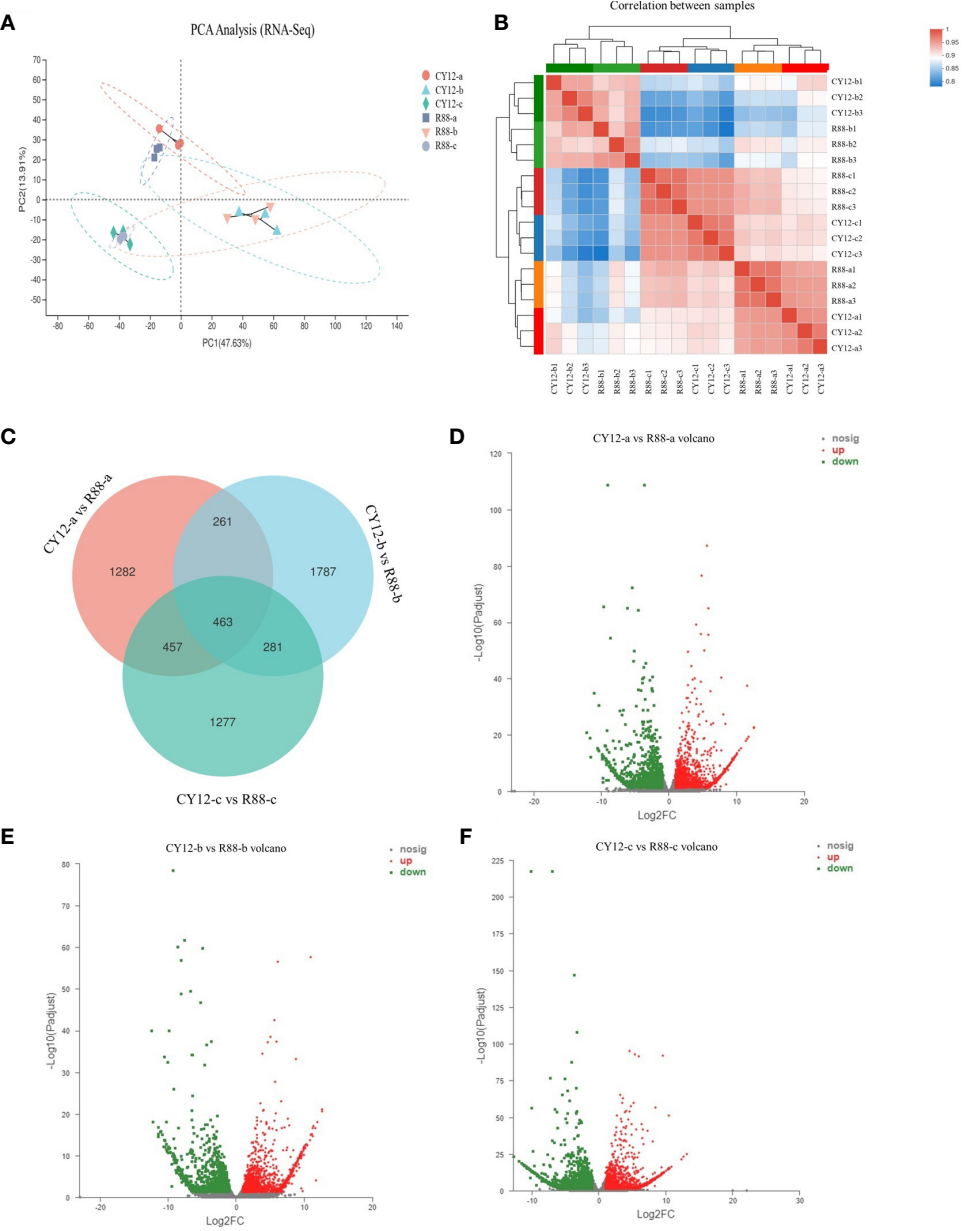
Principal component analysis (PCA) **(A)**, sample to sample correlation analysis **(B)**, Venn diagram **(C)**, and volcano diagram **(D–F)** analysis for differentially expressed genes (DEGs) in wheat after Pst infection. CY12-a and R88-a represent non-inoculated Pst on susceptible and resistant wheat seedlings. CY12-b and R88-b represent inoculated with Pst for 24 h on susceptible and resistant wheat seedlings. CY12-c and R88-c represent inoculated with Pst for 72 h on susceptible and resistant wheat seedlings. In the volcano diagram, red dots represent upregulated DEGs, green dots represent downregulated DEGs, and gray dots represent genes with insignificant differences in expression levels.

Principal component analysis (PCA) showed that the first principal component (PC1) could explain 47.63% of the total variance and distinguish samples based on the time of Pst-CYR34 inoculation, and the second principal component (PC2) could explain 13.91% of the total variance and separate each groups according to the three time points (**Figure 2A**). The heat map of sample correlations (sample to sample clustering) showed that gene expression values among 18 wheat samples were reproducible between the three biological replicates, and batch effects were controlled (**Figure 2B**). The differentially expressed genes (DEGs) were identified by comparing the FPKM values of each gene between CY12 and R88 (at 0-a, 24 hpi-b, and 72 hpi-c) with the criteria of fold change ≥2 and p < 0.05. Then, DEGs involved in the Pst-CYR34 infection process were screened. There were 4,607 DEGs between CY12 and R88 with non-Pst infection, while the number of DEGs changed to 4,834 and 4,456 after 24 and 72 hpi with CYR34 treatment, respectively (**Figure 2C**). There were 2,592 upregulated and 2,015 downregulated genes in R88-a vs CY12-a (**Figure 2D**), 2,721 upregulated and 2,113 downregulated genes in R88-b vs. CY12-b (**Figure 2E**), and 2,252 upregulated and 2,204 downregulated genes in R88-c vs. CY12-c (**Figure 2F**).

### 3.3 GO enrichment and KEGG analysis of DEGs

**Figure 3:**
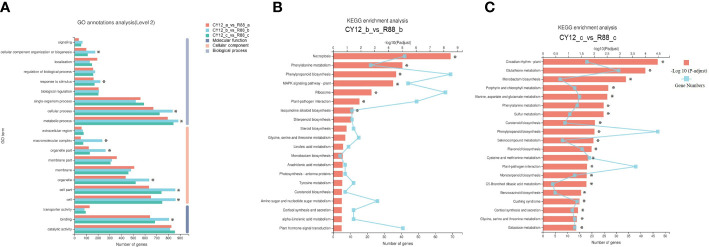
The Gene Ontology (GO) annotations **(A)** and Kyoto Encyclopedia of Genes and Genomes (KEGG) analysis in CY12-b vs R88-b **(B)** and CY12-c vs R88-c **(C)** comparison groups.

The DEGs contributed to the phenotypic differences in Pst-CYR34 infection between CY12 and R88. To identify the major functional terms under Pst-CYR34 treatment, GO enrichment analysis was carried out on the DEGs (**Figure 3A**). GO enrichment results showed that Pst-CYR34-related DEGs were mainly enriched in metabolic process, cellular process, response to stimulus, and biological regulation in the biological process category; cell part, organelle part, and membrane part in the cellular component category; and catalytic activity, binding, and transporter activity in the molecular function category.

DEGs were also enriched in KEGG pathways in this study. At 24 hpi, Pst-CYR34-induced DEGs in CY12 and R88 were notably enriched in necroptosis (ko04217), phenylpropanoid biosynthesis (ko00940), phenylalanine metabolism (ko00360), plant–pathogen interaction (ko04626), and MAPK signaling pathway–plant (ko04016) (**Figure 3B**). A total of 60 DEGs in phenylpropanoid biosynthesis were upregulated, including *PAL*, *POD*, *PTAL*, *COMT*, and *REF1*. Phenylalanine metabolism is the upstream pathway of phenylpropanoid biosynthesis, and most DEGs were also upregulated, including the enzyme encoding genes *amiE* and *MIF*. At 72 hpi, Pst-CYR34-induced DEGs were also enriched in phenylalanine metabolism, phenylpropanoid biosynthesis, flavonoid biosynthesis (ko00941), and plant–pathogen interactions in CY12 and R88 (**Figure 3C**). These results indicate that phenylpropanoid biosynthesis is important for the Pst response in wheat.

### 3.4 Metabolome analysis in wheat after inoculation with Pst

**Figure 4:**
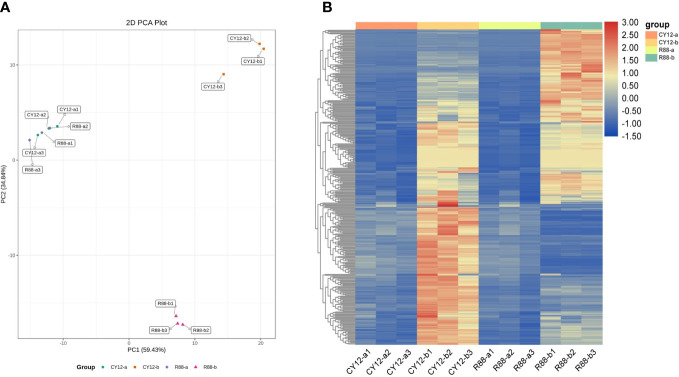
Principal component analysis (PCA) of differentially accumulated metabolites (DAMs) during each Pst inoculation periods in CY12 and R88 **(A)** and the heat map of relative accumulated content of DAMs during each Pst inoculation periods in CY12 and R88 **(B)**.

PCA of metabolome indicated that the PC1 could explain 59.43% of total variance and distinguish wheat seedlings based on the time of Pst-CYR34 inoculation, and the PC2 could explain 34.84% of the total variance (**Figure 4A**). The hierarchical clustering heat map showed each metabolite accumulation values among CY12 and R88 infected by Pst (0 and 24 h) (**Figure 4B**). The results showed that there was no significant difference in the level of metabolite accumulation between (a) R88 and CY12 before Pst inoculation. However, at 24 h after Pst inoculation (b), metabolites in CY12 and R88 accumulated significantly, and CY12 and R88 had their own metabolite content changes in response to Pst infection (**Supplementary Figure S2B**). Therefore, a detailed analysis of these differentially accumulated metabolites was carried out in this experiment.

**Figure 5:**
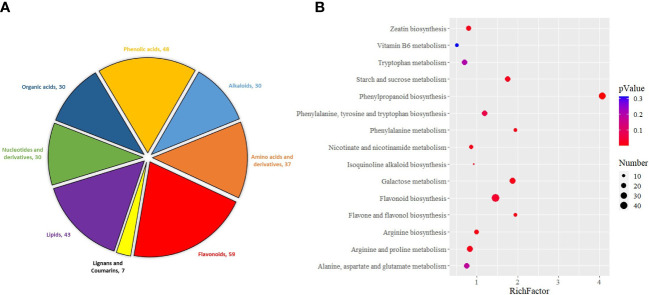
Classification of metabolites in Pst-resistant and Pst-susceptible wheat seedlings at different periods **(A)** and KEGG enrichment analysis of differential accumulation metabolites **(B)**.

First, differential accumulation metabolites (DAMs) identified after Pst inoculation were classified. Among them, flavonoids (59) and phenolic acids (48) account for 31.94% of the total metabolites (**Figure 5A**). The results showed that flavonoids and phenolic acids in wheat played a very important role in the resistance to Pst infection. Then, these metabolites were enriched by KEGG function. It turns out that these DAMs are mainly enriched in phenylpropanoid biosynthesis, flavonoid biosynthesis, starch and sucrose metabolism, phenylalanine, tyrosine, and tryptophan biosynthesis (**Figure 5B**). This result was also significantly related to the types of metabolites, suggesting that these pathways are also the main factors involved in wheat resistance to Pst.

### 3.5 Candidate gene cloning and VIGS verification

In order to further explore the relationship between phenylpropanoid, flavonoid biosynthesis, and lignin accumulation to Pst resistance in wheat, *TaPAL*, an upstream regulatory gene regulating flavonoid and lignin synthesis, was selected as a candidate gene for cloning and functional analysis based on gene expression and metabolite accumulation content. To verify the reliability of RNA-Seq data, we performed RT-PCR analysis of *TaPAL* gene (*TraesCS6B02G258400*). The results showed that CY12 and R88 had the same expression level of *TaPAL* gene before inoculation with Pst. However, the expression level was significantly increased 24 h after inoculation, and R88 was significantly higher than CY12 (**Supplementary Figure S2A**). Next, we designed primers to clone this candidate gene and to compare with Chinese spring sequences, in which the comparison rate was >97.1% and contains PLN02457 conserved domain (**Supplementary Figure S2B**).

**Figure 6:**
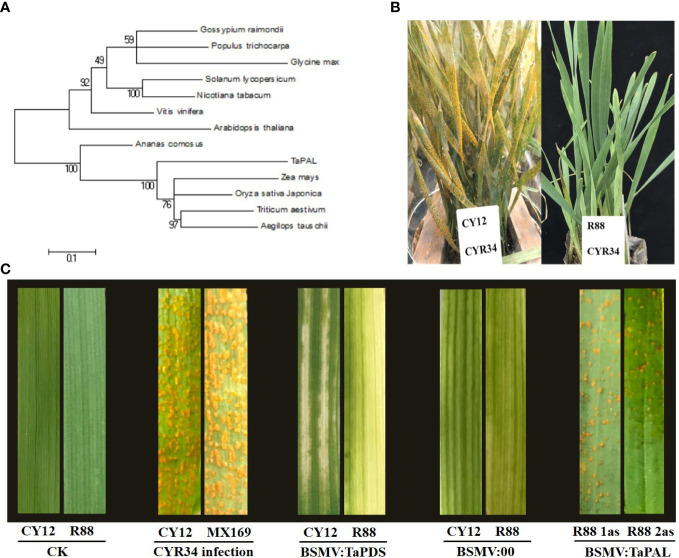
Phylogenetic analysis of *TaPAL* in wheat and other plants **(A)**, phenotypes of CY12 and R88 after inoculation with Pst-CYR34 at 28 days **(B)**, and short-time gene silencing of *TaPAL* by VIGS for gene function verification **(C)**. CK, not inoculated Pst-CYR34 (negative control); BSMV : TaPDS, silence TaPDS gene; BSMV:00, unconnected gene fragment (positive control); BSMV : *TaPAL*, silence TaPAL gene.

The phylogenetic analysis revealed a close relationship of *TaPAL* to PAL proteins in *Zea mays*, *Oryza sativa*, *Aegilops tauschii*, and other plant species (**Figure 6A**). In this experiment, the phenotypes of CY12 and R88 seedlings were observed at 28 days after inoculation with Pst-CYR34. Among them, CY12 seedling leaves were basically covered by stripe rust spores, while R88 was almost immune to stripe rust (**Figure 6B**). Two specific gene fragments (1 as and 2 as) were designed to specifically silence the *TaPAL* gene in wheat. Compared with CK, the 2-week-old wheat leaves faded after inoculation with BSMV : TaPDS in CY12 and R88 (**Figure 6C**). The results suggest that *TaPDS* was successfully silenced in wheat seedlings. The virus leaf with empty vector and target gene showed striped chlorotic symptoms (**Figure 6C**), which indicate that the *TaPAL* gene silencing vector was also successfully constructed in this study. After 20 days of inoculation with Pst-CYR34, CY12 and MX169 were very susceptible to disease and a large number of spore piles appeared in the leaves, while R88 was a near-immune wheat line and almost no spore piles were seen in the leaves (**Figure 6C**). Compared with WT (R88 without gene silencing), R88 1as and R88 2as inoculated with Pst-CYR34 displayed susceptibility in BSMV : TaPAL-silenced lines, which leaves appeared a small amount of spore piles (**Figure 6C**). These results suggest that *TaPAL* is associated with Pst-CYR34 resistance in R88 wheat, which can positively regulate the resistance response in wheat by regulated phenylpropanoid biosynthesis and lignin biosynthesis.

### 3.6 Combined transcriptome and metabolome analysis in wheat to resistant Pst-CYR34

**Figure 7:**
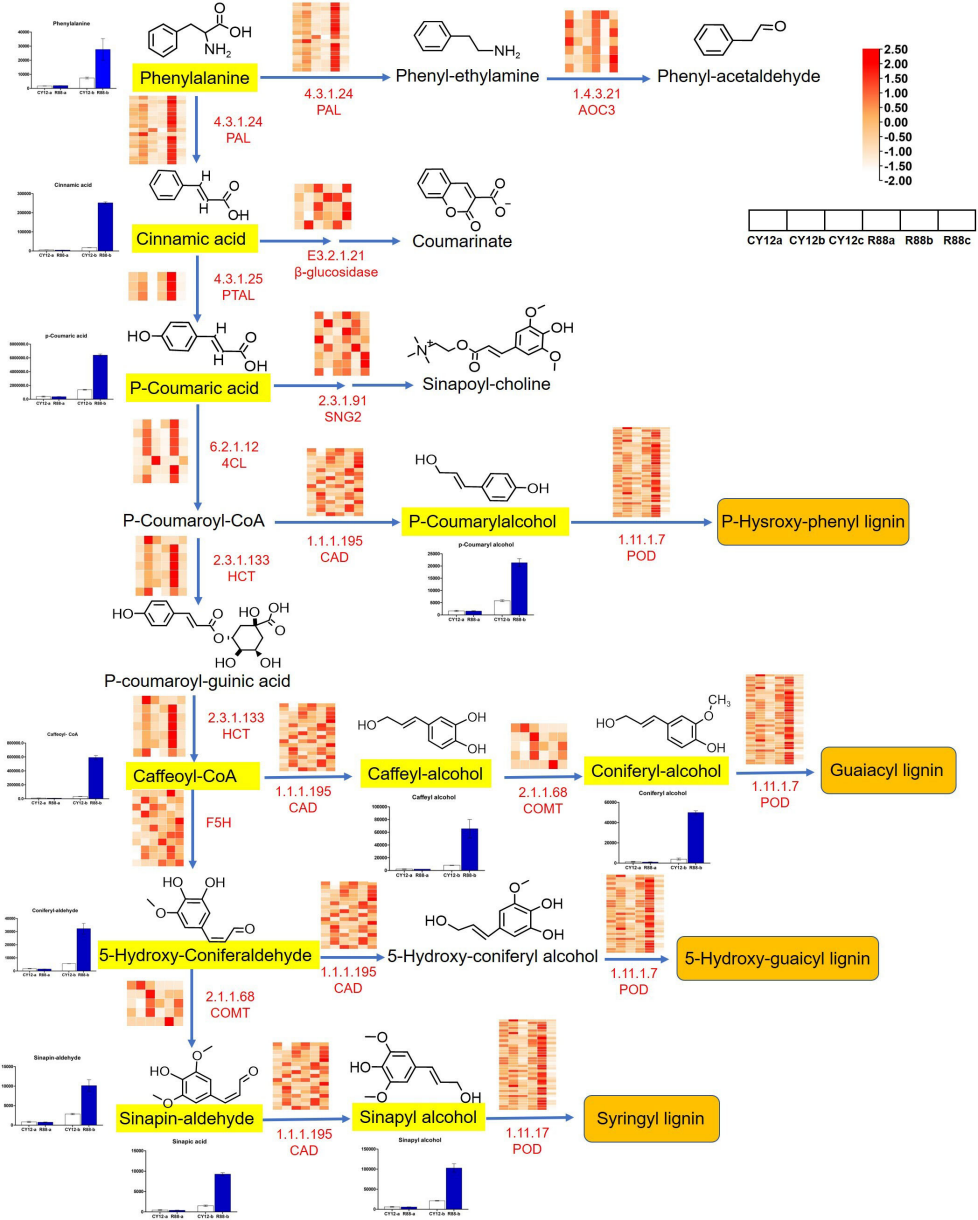
Analysis of DEGs and differentially accumulated metabolites (DAMs) in phenylpropanoid biosynthesis pathway flow to lignin synthesis after inoculation of Pst in wheat. The black letters represent metabolites produced in the pathway, with the chemical formula for the metabolite at the top. The bar chart shows the relative content changes of the corresponding metabolites during different periods of Pst inoculation. The small heat map shows the changes in gene expression marked in red letters during different periods of Pst inoculation. In the color block bar, red represents upregulated gene expression, while white represents downregulated gene expression.

Combined analysis of transcriptome and metabolome data showed that the Pst-induced DEGs and metabolites involved in phenylpropanoid biosynthesis were examined in this study. Compared with CY12, most DEGs of phenylpropanoid biosynthesis pathway were upregulated in L58 after Pst inoculation at 24 h (**Figure 7**). In this study, phenylpropanoid was biosynthesized from phenylalanine, followed by different steps of the pathway catalyzed by four types of enzymes: ammonia lyase (PAL), hydroxycinnamoyl transferase (HCT), cinnamyl alcohol dehydrogenase (CAD), and E1.11.1.7. Finally, the pathway produced three types of monolignols that polymerize to form lignin: p-hydroxyphenyl (H), guaiacyl (G), 5-hydroxy-guaicyl, and syringyl (S) lignin. The expression levels of DEGs in this pathway were illustrated using a heat map and estimated by log2 (fold change) (**Figure 7**). Although there were some cases of downregulation or non-significant upregulation for some genes at different time points, most of the four types of genes in this pathway were upregulated in R88 within 24 hpi. Generally, the upregulation of phenylpropanoid biosynthesis genes began at 24 hpi or earlier.

Based on the upregulation of genes related to the phenylpropanoid biosynthesis pathway, we determined lignin biosynthesis-related metabolites by LC-MS/MS in CY12 and R88 at 24 hpi. Metabolome analysis showed that a total of 11 lignin-related metabolite compounds were detected in wheat, including phenylalanine, cinnamic acid, p-coumaric acid, p-coumarylalcohol, caffeoyl-coA, caffeyl-alcohol, coniferyl-alcohol, 5-hydroxy-coniferaldehyde, sinapin-aldehyde, and sinapyl-alcohol (**Figure 7**). Compared with CY12, the content of these metabolites was significantly increased in R88 at 24 hpi. All of these compounds had a >1.5-fold change in content. This result is consistent with that of gene expression in the transcriptome.

## 4 Discussion

Plant pathogens are major factors affecting crop yield and quality worldwide. Plants have evolved pathogen-associated molecular pattern-triggered immunity (PTI) and damage-associated molecular patterns that trigger immunity and effector-triggered immunity (ETI), which generate resistance gene products to specifically recognize the effectors released from the invader and to confer disease resistance (Tang et al., 2017). Previous studies have shown that proline act as osmo-regulator to biotic or abiotic stress, which can regulate osmolytes accumulation to scavenge reactive oxygen species in response to these different stresses (Li et al., 2017). In the present study, our results indicated that the proline content were significantly increased in wheat seedlings after Pst infection. Similar to previous studies, proline significantly accumulated in tomato, eggplant, and certain mustard varieties by blight fungi infection (Kumari et al., 2017; Attia et al., 2022). The MDA content increased significantly by Pst infection at 24 and 72 h in both CY12 and R88 wheat seedlings, which can cause intracellular oxidative stress to severe disturbance in the plant cell (Badawy et al., 2021). However, the results of this experiment showed that the increased level of MDA in R88 was significantly lower than that in CY12 after Pst infection. This result indicates that Pst-resistant wheat plants can minimize the damage to plant cells due to lower MDA accumulation content. In addition, this experiment found that CAT, PAL, and POD enzyme activities are also related to plant resistance. The results of gene expression levels corresponding to these physiological indexes were consistent in the transcriptome profile. The results showed that most of the DEGs were highly expressed after inoculation with CYR-34. In addition, we found that those genes together with other genes also participate in the activation and response of disease-resistant pathways. These immune strategies trigger downstream responses, including the accumulation of secondary metabolites to activate defense systems (Wu et al., 2014).

Phenylpropanoid biosynthesis metabolism is the most important secondary metabolic pathway, playing a fundamental role in plant defense against biotic (invading pathogens) and abiotic stresses (La Camera et al., 2004). The main branches of the phenylpropanoid pathway lead to the synthesis of lignin and flavonoids. Although certain studies have provided evidence to suggest that lignin, flavonoid, and phenolic compounds play important roles during the plant defense response to pathogens, few studies have analyzed the overall transcriptome and metabolome changes in phenylpropanoid biosynthesis genes or related metabolites in wheat. The present results indicated that the total gene expression and metabolite accumulation profiles of the phenylpropanoid biosynthesis pathway were responsive to Pst-CYR34, which is consistent with the results of our previous studies (Liu et al., 2022). As a result, the study focused on the phenylpropanoid biosynthesis pathway with an emphasis on the synthesis and deposition of lignin because most of the enriched DEGs and related metabolites were upregulated in this pathway. Previous studies have reported that induced lignification is one of several plant defense responses to wounding and to viral or microbial attack (Bhuiyan et al., 2009). In infected plants, lignification and deposition of lignin are part of plant cell wall reinforcement and are important processes in the response of plants to restrict pathogen invasion (Kauss et al., 1993) and provide a physical barrier to limit pathogen colonization (Bonello and Blodgett, 2003).

Induced lignification around infection centers is generally accompanied by the increased activity of a number of enzymes. Genetic evidence for the role of lignin-associated defense is rare, however, either because of the indispensability of lignin for the plant form. In this study, DEGs encoding the enzymes involved in the lignin pathway showed upregulated expression, which indicated the putative role of lignin in the wheat defense response (Liu et al., 2022). The activities of enzymes involved in the biosynthesis of lignin were measured in this study. POD is involved in the polymerization of monolignols into lignin and reinforcement of the cell wall after pathogen attack and wounding (Marjamaa et al., 2009). Phenylalanine ammonia-lyase (PAL) plays an essential role in the phenylpropanoid pathway and has been reported to be responsive to both biotic and abiotic stresses, including pathogen attack, wounding, cold, UV stress, and other stress conditions (Huang et al., 2010). Consistent with previous reports, PAL and POD activities increased with localized lignin deposition (Nicholson and Hammerschmidt, 2003; Caldo et al., 2004) after Pst-CYR34 inoculation in both susceptible and resistant wheat. However, compared with resistant plants (R88), the inoculated susceptible plants (CY12) showed an obviously slower increase and lower enzyme activities. This result suggests that lignin may be the active form of lignification in HR to protect cells against fungal invasion (Bhuiyan et al., 2009). Together with the gene expression profiles, we speculated that the most significant difference between susceptible and resistant plants was whether the plants could activate the pathway in a timely and efficient manner.

The lignin biosynthetic pathway is initially induced in both resistant and susceptible wheat following rust infection (Moerschbacher et al., 1988). In this study, the metabolites of the lignin biosynthesis pathway were investigated in both resistant and susceptible wheat seedlings after Pst-CYR34 infection. Pst-CYR34 increased the total lignin content in wheat and to a lesser degree for CY12 than R88, which suggested that lignification may be a mechanism through which wheat restricts the pathogen. An increase in the lignin content was determined in the inoculated resistant wheat (R88) compared with the control at both 24 and 72 h after Pst infection. The 11 lignin-related compounds determined by LC-MS/MS also showed upregulation in R88 compared with CY12 at 24 hpi. Overall, the resistant wheat line displayed increased lignin concentrations and was speculated to play a vital role in the wheat defense response. In accordance with these reports, this result suggested that increased lignification or lignin biosynthesis is critically important for wheat to defend against pathogen invasion.

PAL is a key enzyme in the phenylpropanoid pathway, which is involved in the improvement of resistance plants (Zhang and Liu, 2015; Lu et al., 2019). *PAL* gene expression can be induced at the transcriptional level under the stress of exogenous signal compounds (mechanical damage, bacteria, and viruses), and PAL activity increases rapidly to activate phenylpropane metabolism in the defense system (Leyva et al., 1992; Pina and Errea, 2008). In this study, we have found that the *TaPAL* plays an important role involved in resistance response by regulating the production of lignin in wheat. After transient VIGS silencing *TaPAL* gene expression, Pst-resistant wheat line R88 showed symptoms of disease. These results suggest that *TaPAL* gene also plays an important role in wheat resistance to Pst. Previous studies have shown that PAL is also important for pathogen-induced SA formation and phenolic phytoalexins biosynthesis in plants (Huang et al., 2010; He et al., 2020). Therefore, the results of this experiment speculated that *TaPAL* gene not only regulates the synthesis of lignin in wheat but also participates in the synthesis of phenolic phytoalexins and SA, so as to play a dual defense role in wheat after Pst infection.

## 5 Conclusion

To better understand the possible molecular mechanisms of plant–pathogen interactions and to improve plant disease resistance, molecular biology and bioinformatic technology have recently been developed to achieve these goals and cultivate wheat stripe rust resistance varieties. It is very important to mine resistance genes, which can improve wheat resistance to Pst and combine with other resistance-related genes to activate certain pathways to defend against the invasion of pathogenic microorganisms. In responding to pathogen (Pst-CYR34) invasion, wheat plants sense external signals in a timely fashion, and then, signal transduction is conducted by signaling pathways, plant defense-related gene expression, and cell wall reinforcement in wheat to an immune response. In the resistant wheat line R88, transcripts of lignin biosynthesis genes were significantly expressed at 24 hpi, which led lignin content accumulated in wheat to be a defense against Pst infection. By combining RNA-seq, LC-MS/MS and biochemical data, it was speculated that DEG expression levels, metabolite accumulation, enzyme activities, and total lignin contents were increased after Pst inoculation in wheat. At the same time, the function of *TaPAL*, a key enzyme in the phenylpropanoid biosynthesis pathway, was analyzed in the present study, which plays an important role in the regulation of lignin biosynthesis. The VIGS verification results show that *TaPAL* gene expression positively regulated Pst resistance in wheat. Therefore, a critical role for *TaPAL* expression level and lignin content was deduced to contribute to wheat disease resistance.

## Data availability statement

The datasets presented in this study can be found in online repositories. The names of the repository/repositories and accession number(s) can be found in the article/**Supplementary Material**

## Author contributions

RL and XL contributed to the methodology, analyzed RNA-Seq and LC-MS/MS data, gene clone, draw figures, and wrote the draft-manuscript. RL and XW performed experimental physiological index detection. LY analyzed RNA-Seq and LC-MS/MS data. JC, YD and WW reviewed and edited the manuscript. YW and RL contributed to the conceptualization, resources, supervision, and funding acquisition. All authors contributed to the article and approved the submitted version.
