# Supplementary material for: Integrative analysis of the multi-omics reveals the stripe rust fungus resistance mechanism of the TaPAL in wheat
Source: Front Plant Sci. 2023 Jun 5;14:1174450. doi: 10.3389/fpls.2023.1174450 (PMC10277697; doi:10.3389/fpls.2023.1174450)
Supplement: Supplementary file 1 [file Table_1.docx]

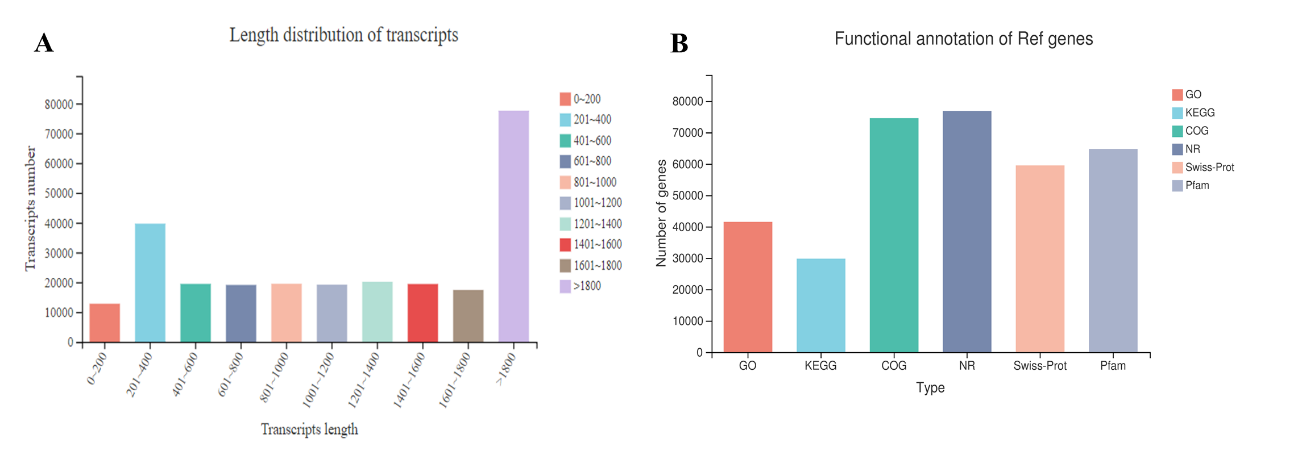


Figure S1. Statistical analysis the length distribution of transcripts form RNA-Seq data (A), and the functional annotation of reference (Ref) genes in wheat (B).


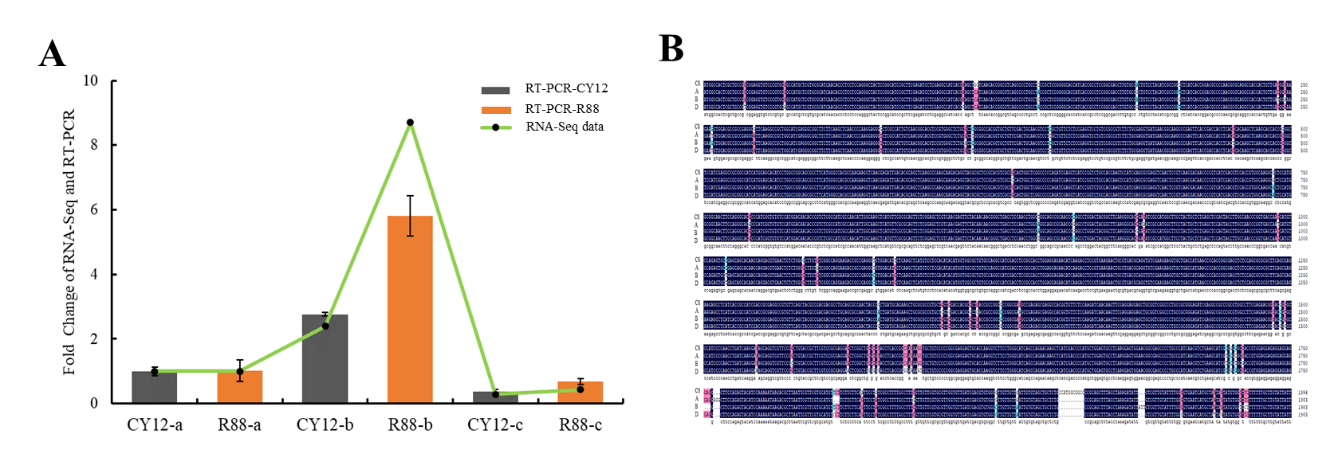


Figure S2. The gene expression level of TaPAL was analyzed by RT-PCR (A). Sequence comparison of three alleles of *TaPAL* gene in wheat line and Chinese spring reference genome (B).
