# Supplementary material for: Integrative analysis of the multi-omics reveals the stripe rust fungus resistance mechanism of the TaPAL in wheat
Source: Front Plant Sci. 2023 Jun 5;14:1174450. doi: 10.3389/fpls.2023.1174450 (PMC10277697; doi:10.3389/fpls.2023.1174450)
Supplement: Supplementary file 2 [file Table_2.docx]

Table S1. List of all primers used in this study.

| Name | F primer (5’-3’) | R primer (5’-3’) | Size (bp)/TM |
| --- | --- | --- | --- |
| L6A1 | TCCATCAGGGAATCTCAAGG | ACTGCCTAGTGGGTTGGTTG | 1418/55℃ |
| L6A2 | CTGTTCTCTCCGAGGTCCTG | GAAACGGAGGGAGCATTGTA | 1641/56℃ |
| L6B1 | CATGCGTCCCCGATAAACTA | CATGAGCTTGAGGATGTCCA | 1223/55℃ |
| L6B2 | AAGCCCAAGCAAGACAGGTA | TAGGTAAAGCTGCGGCAGAG | 1320/55℃ |
| L6D1 | TAGATCCCGTGCTTCTGCTT | GAACGGACACCACAGTTCCT | 1341/57℃ |
| L6D2 | TCACCAAGCTCATCAACACC | ACTCTGGAAGCCTCCTCCTC | 1656/58℃ |
| V-TaPAL1as | CCTTAATTAAGGTCAAGCTCATGTCCTCCACA | TTAATTAAGGGGGTCGTCGGCGTAGCTGAAC | 221/60℃ |
| V-TaPAL2as | CCTTAATTAAGGCATCAGCCAGAACAAGCTCA | TTAATTAAGGGGGCAAAAGGCGATAGGAACTG | 202/60℃ |
| qTaPAL | CCACTGTTGACGGGAAGAAT | CAGGACCTCGGAGAGAACAG | 185/56.5℃ |
| TaGAPDH | AAGGCTGTTGGCAAGGTG | GTGGTCGTTCAGAGCAATCC | 192/59℃ |
